# Opportunistic sampling from the near‐threatened Alexandrine parakeet uncovers genomes of a novel parvovirus and beak and feather disease virus

**DOI:** 10.1111/avj.13442

**Published:** 2025-03-29

**Authors:** S Sarker, N Klukowski, S Talukder, A Athukorala, MJ Uddin

**Affiliations:** ^1^ Biomedical Sciences and Molecular Biology, College of Medicine and Dentistry James Cook University Townsville Queensland 4811 Australia; ^2^ Australian Institute of Tropical Health and Medicine, James Cook University Townsville Queensland 4811 Australia; ^3^ Department of Microbiology, Anatomy, Physiology and Pharmacology School of Agriculture, Biomedicine and Environment, La Trobe University Melbourne Victoria Australia; ^4^ College of Science and Engineering, James Cook University Townsville Queensland 4811 Australia; ^5^ School of Veterinary Medicine, Murdoch University Perth Western Australia 6150 Australia; ^6^ Centre for Biosecurity and One Health, Harry Butler Institute, Murdoch University Perth Western Australia 6150 Australia

**Keywords:** genomics, metagenomics, parrot, phylogenetics, virome

## Abstract

Birds are known to harbour a wide range of pathogenic viruses, including the beak and feather disease virus (BFDV; species, *Circovirus parrot*), which poses a significant threat to the conservation of endangered avian species. This study reports the genomic identification and characterisation of a novel psittaciform chaphamaparvovirus (PsChPV‐6) and BFDV, sequenced from the faecal samples of healthy Alexandrine parakeets (*Psittacula eupatria*). PsChPV‐6 is a linear, single‐stranded DNA virus consisting of 4232 nucleotides (nt) with a high A + T content and five predicted open reading frames (ORFs). Key proteins encoded by PsChPV‐6, such as the nonstructural protein 1 (NS1) and major capsid protein VP1, demonstrate strong sequence similarities to other avian parvoviruses, with conserved motifs in NS1 crucial for viral replication. The presence of a previously uncharacterised ORF1 region suggests strain‐specific viral features that warrant further exploration. BFDV is a circular single‐stranded DNA virus in the *Circoviridae* family and was also identified in the samples. Phylogenetic analysis positioned PsChPV‐6 within the *Chaphamaparvovirus* genus, closely related to parvoviruses from diverse avian species, whereas BFDV was grouped with strains from Australian cockatoos and other nonpsittacine birds, suggesting potential cross‐species transmission. These findings contribute to a deeper understanding of the genetic diversity and evolutionary dynamics of these viral pathogens in bird populations, underscoring the importance of ongoing surveillance to evaluate their ecological and veterinary impacts.

**Abbreviations**
AGRF: Australian genome research facility
BFDV: beak and feather disease virus
CTCPaV: chestnut teal chaphamaparvovirus
cDNA: complementary DNA
EDTA: ethylenediaminetetraacetic acid
IUCN: International Union for Conservation of Nature
ORF: open reading frames
PBFD: psittacine beak and feather disease
PsChPV‐6: psittaciform chaphamaparvovirus
ssDNA: single‐stranded DNA

Viral disease can greatly affect the conservation efforts for bird species. In recent years, numerous species have been classified as endangered (EN), critically endangered (CE), vulnerable or near‐threatened by the International Union for Conservation of Nature (IUCN). According to the IUCN, the number of EN and CE bird species increased from 403 in 1996 to 773 in 2016, out of a total of 11,121 species.^1^ This trend underscores the importance of surveilling both known and undiscovered viruses that circulate among wild and captive birds, from both conservation and economic viewpoints.

The Alexandrine parakeet (*Psittacula eupatria*), a prominent member of the parrot family, is native to regions across South and Southeast Asia. Recognisable by its vibrant green feathers, bright red beak and maroon shoulder patch, despite its ability to thrive in diverse habitats, ranging from forests to urban areas, the Alexandrine parakeet is currently listed as Near‐Threatened by the IUCN.^2^ Key threats to this species include habitat destruction due to deforestation, agricultural activities and urban expansion, as well as illegal trapping for the pet trade.^2^ With its population in decline in many areas, urgent conservation measures are needed to protect this iconic bird. In addition, investigating unknown viruses that may present in the Alexandrine parakeet is also necessary.

Metagenomic studies in various animal samples enable the detection and characterisation of numerous viruses.^3^, ^4^, ^5^, ^6^, ^7^, ^8^, ^9^, ^10^ Therefore, this study employed metagenomic techniques to detect viruses present within the sampled Alexandrine parakeets, thereby expanding our knowledge on viruses present within captive pet psittacines and enabling better surveillance on potential viruses.

## Materials and methods

### Sampling and ethical consideration

Faecal samples were obtained from three healthy Alexandrine parakeets kept together in a pet shop located in Victoria, Australia. The samples were gathered from food trays during routine husbandry practices by us, ensuring no direct contact with the birds. The Alexandrine parakeets were kept in a separate case; however, several other bird species, including African grey parrots, Indian ring‐necked parakeets, yellow canaries and rainbow lorikeets, were also housed in the shop. They were then stored at −80°C until further analysis. The La Trobe University Animal Ethics Committee granted approval for the use of these diagnostic materials in publication.

### Virus enrichment and virus nucleic acid extraction

After removing potential contaminants such as host cells, bacteria, food debris and free nucleic acids from the faecal samples, viral particle enrichment was carried out using previously established protocols,^7^, ^11^ with slight modifications. In short, the faecal material was aseptically resuspended and thoroughly homogenised in sterile phosphate‐buffered saline (PBS) at a 1:10 ratio. The mixture was then centrifuged at 17,000 g for 3 min at room temperature (RT). The resulting supernatant was passed through a 0.80 μm syringe filter, and the filtrate was subjected to further processing. Ultracentrifugation of the samples was performed at 178,000 g for 1 h at 30 PSI and 4°C using a Hitachi Ultracentrifuge CP100NX. After discarding the supernatant, the pellet was resuspended in 130 μL of sterile PBS. To eliminate residual nucleic acids, the filtrates were treated with 2 μL of benzonase nuclease (25–29 U/μL, >90% purity, Millipore) and 1 μL of micrococcal nuclease (2,000,000 gel U/mL, New England Biolabs), followed by incubation at 37°C for 2 h. The nuclease activity was halted by adding 3 μL of 500 mM EDTA. Viral nucleic acids were extracted using the QIAamp Viral RNA Mini Kit (Qiagen, Valencia, CA, USA) without the use of carrier RNA, allowing for the simultaneous extraction of both viral DNA and RNA. The quantity and quality of the extracted nucleic acids were assessed using a Nanodrop and an Agilent TapeStation (Agilent Technologies, Mulgrave, VIC, Australia) at the Genomic Platform, La Trobe University.

### Next‐generation sequencing

Before library construction, the extracted RNA underwent cDNA synthesis, followed by amplification using the Whole Transcriptome Amplification Kit (WTA2, Sigma‐Aldrich, Darmstadt, Germany) in accordance with the manufacturer's guidelines. The amplified PCR products were purified using the Wizard® SV Gel and PCR Clean‐Up Kit (Promega, Madison, WI, USA). The concentration and quality of the purified products were evaluated using the Qubit dsDNA High Sensitivity Assay Kit and a Qubit Fluorometer v4.0 (Thermo Fisher Scientific, Waltham, MA, USA).

Library construction for pooled samples was carried out using the Illumina DNA Prep Kit (Illumina, San Diego, CA, USA), according to the manufacturer's protocol, with an initial input of 250 ng of DNA, as quantified by the Qubit Fluorometer v4.0 (Thermo Fisher Scientific). The Australian Genome Research Facility (AGRF) in Melbourne, Australia, assessed the quality and quantity of the prepared library. The library was normalised and pooled in equimolar amounts. Before sequencing, the quality and concentration of the final pooled library was reassessed using the same methods. Cluster generation and sequencing was performed at AGRF using the Illumina® NovaSeq platform, with 150‐bp paired‐end reads, according to the manufacturer's instructions.

### Bioinformatic analyses

Sequencing data were analysed using an established pipeline^12^, ^13^, ^14^, ^15^ in Geneious Prime (version 2023.1.1, Biomatters, New Zealand). Initially, 36.77 million raw reads were processed to eliminate Illumina adapters, ambiguous base calls and low‐quality reads (quality trimming threshold set to 0.05; ambiguous nucleotides trimmed up to 15). The cleaned reads were then mapped against the chicken genome (*Gallus gallus*, GenBank accession no. NC_006088) to remove any potential host DNA contamination. Additionally, the reads were mapped to the *Escherichia coli* genome (GenBank accession no. U00096) to exclude bacterial contamination. The unmapped, filtered reads were then used for de novo assembly via the SPAdes assembler (version 3.10.1),^16^ using the ‘careful’ setting on the LIMS‐HPC system, a high‐performance computer dedicated to genomic research at La Trobe University. The assembled contigs were compared against the nonredundant nucleotide and protein databases on GenBank using BLASTN and BLASTX,^17^ respectively, with an *E*‐value cutoff of 1 × 10^−5^ to minimise false positives. Contigs with significant hits to bacteria, eukaryotes or fungi were filtered out to eliminate nonviral sequences. Viral contigs longer than 300 nucleotides were selected for further functional analysis in Geneious Prime (version 2023.1.1). The average coverage of viral contigs were calculated using the cleaned raw reads in the same software.

### Functional annotations

The complete viral genomes assembled in this study were annotated following established protocols^6^, ^7^ using Geneious Prime (version 2023.1.1, Biomatters, Ltd., Auckland, New Zealand). Viral taxonomy was identified through comparative analyses using GenBank's BLASTN, BLASTX and BLASTP, with the highest match being selected based on specific criteria (*E*‐value < 0.0). Open reading frames (ORFs) within the viral genomes were annotated by comparing them against NCBI's database. Additionally, the ORFs were compared with conserved domain databases (NCBI, Bethesda, MA, USA).^17^ All software settings were left at default unless otherwise noted.

### Comparative genomics and phylogenetic analyses

Genomic comparison of the newly sequenced viral genomes was carried out using Geneious Prime (version 2023.1.1). Sequence similarities between the selected viral sequences and representative viral genomes were determined by performing MAFFT alignment (L‐INS‐I) within Geneious Prime (version 2023.1.1, Biomatters, Ltd., Auckland, New Zealand).

For phylogenetic analysis, representative parvoviral genomes or complete NS1 gene sequences were obtained from GenBank, and a phylogenetic tree was constructed in Geneious Prime (version 2023.1.1). The amino acid sequences of the NS1 protein‐coding genes were aligned using the MAFFT L‐INS‐I algorithm in Geneious Prime (version 7.388).^18^ The phylogenetic tree was built using RAxML with the Gamma Blosum62 protein model and 1000 bootstrap replicates in Geneious Prime (version 2023.1.1). For BFDV, the complete genome sequences were aligned using MAFFT (version 7.450) with the G‐INS‐I algorithm in Geneious Prime (version 23.1.1), and a maximum likelihood (ML) tree was generated with 500 replicates. The resulting phylogenetic trees were visualised using FigTree v1.4.4.

## Results

### Genomic features of the detected novel PsChPV‐6

The genome of the novel psittaciform chaphamaparvovirus 6 (PsChPV‐6) was identified as a linear single‐stranded DNA (ssDNA) molecule comprising 4232 nucleotides (nt), with a sequencing depth of 144.2×. Its genomic structure closely resembled that of other known *Parvoviridae* family members. The genome had an A + T content of 60.7% and a C + G content of 39.3%, aligning with the typical base composition of parvoviruses. Structurally, PsChPV‐6 contained two major predicted ORFs, likely encoding the replication initiator protein (NS1) and the major capsid protein (VP1), consistent with the characteristic features of parvoviral genomes.

### PsChPV‐6 shows a high genetic diversity

The PsChPV‐6 genome contains five ORFs, which were comparatively analysed via BLASTX and BLASTP tools.^17^ Four of the ORFs displayed significant similarities in protein sequences (*E*‐value ≤ 10^−5^) with established parvoviruses. However, the 5′ ORF1, spanning 213 nt, did not reveal any significant protein matches in BLAST searches using its putative amino acid sequence.

The nonstructural protein 1 (NS1) ORF of PsChPV‐6 was 2010 nt long, showing the highest amino acid sequence identity with psittacine parvovirus 1 (50.67% identity, 99% query coverage, GenBank accession no. YP_010805270.1). It was followed by psittaciform chaphamaparvovirus 3 (54.55% identity, 73% query coverage, GenBank accession no. AYC49708.1). As seen in other parvoviruses, the full‐length NS1 gene of PsChPV‐6 comprises 669 amino acids (AA) and encodes a helicase, including conserved ATP‐ or GTP‐binding motifs: Walker A loop (GPxNTGKT/S; _317_ **GP**S**NTGKS**_324_), Walker B (xxxWEE; _355_IGI**WEE**_360_) Walker B′ (KQxxEGxxxxxPxK; _372_ **KQ**VL**EG**MTCSI**P**I**K**_385_) and Walker C (PxxxTxN; _396_ **P**IFI**T**T**N**_402_) AA motifs. The NS1 protein contains two conserved replication initiator (endonuclease) motifs, xxHuHxxxx (IF_108_ **H**V**H**_110_CLLR) and YxxK (_166_ **Y**MM**K**_169_) (conserved amino acids are indicated in bold letters, and “u” indicates a hydrophobic residue). The nonstructural protein (NS) 2 and 3 encoding ORFs, measuring 714 and 441 nt, respectively, showed the highest amino acid identity with galliform chaphamaparvovirus 17 (54.82% identity, GenBank accession no. UOH27079.1) and galliform chaphamaparvovirus 11 (50.0% identity, GenBank accession no. UOH27053.1), respectively.

The major 3′ ORF, which spans 1500 nucleotides, encoded a protein homologous to the VP1 capsid protein of the *Parvoviridae* family. At the amino acid level, the VP1 protein of PsChPV‐6 shared the highest identity with the capsid proteins of Ara ararauna chaphamaparvovirus and psittaciform chaphamaparvovirus 5, with protein identities exceeding 54% (GenBank accession nos. QTE04011.1 and WOX03043.1, respectively).

### Genome of the detected BFDV

The genome of the detected BFDV was a circular ssDNA molecule, comprising 2009 nt, with a sequencing depth of 35.73×. Its genomic structure closely resembled that of other known members of the *Circoviridae* family. Structurally, BFDV contained two primary predicted ORFs: one encoding a replication‐associated protein (Rep) and the other encoding a capsid protein (Cap). The Rep and Cap ORFs, measuring 873 and 744 nt, respectively, exhibited the highest amino acid sequence identity with BFDV sequences from *Nymphicus hollandicus* for Cap (99.66% identity, GenBank accession no. ABO65371.1) and *Cacatua tenuirostris* for Rep (98.38% identity, GenBank accession no. AHX56441.11).

### Evolutionary relationships

**Figure 1:**
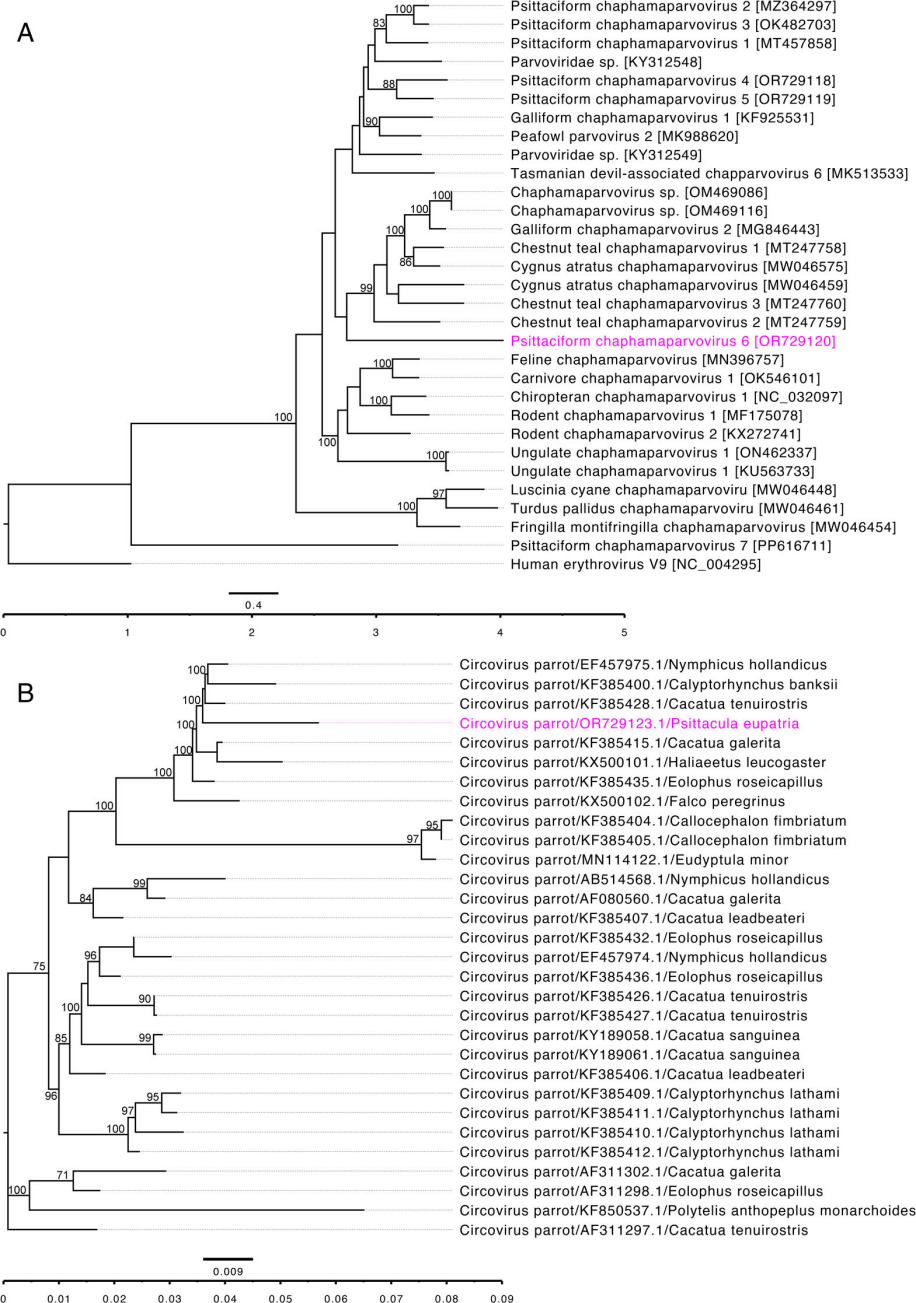
The phylogenetic tree illustrates the potential evolutionary relationships of selected parvoviruses (A) and BFDV (B). (A) Amino acid sequences of the complete NS1 gene were extracted individually from the PsChPV‐6 and various selected parvovirus genomes, then aligned with MAFFT (version 7.450), using G–INS–I (scoring matrix BLOSUM62; gap open penalty 1.53; offset value 0.123) in Geneious Prime (version 23.1.1, Biomatters, Ltd., Auckland, New Zealand). ML tree was generated in Geneious Prime with 1000 replicates using human erythrovirus V9 as the outgroup. Sequence diversities are indicated as substitutions per site on the branches, and the labels at the branch tips represent the original parvovirus species names along with their GenBank accession numbers in parentheses. The PsChPV‐6 sequence analysed in this study is highlighted in pink. (B) Selected complete genome sequences of BFDV were aligned with MAFFT (version 7.450), using G–INS–I in Geneious Prime (version 23.1.1). ML tree was generated in Geneious Prime with 500 replicates. Trees were visualised using FigTree v1.4.4 and tip labels were aligned. Sequence genetic distance is indicated as substitutions per site on the branches in branch labels, and the labels at the branch tips indicate the representative virus species names followed by their GenBank accession and host. The BFDV sequence analysed in this study is highlighted in pink. An automatic scale bar and scale axis were added. Bootstrap values at the nodes are indicated as percentages (bootstrap value lower than 70% was removed from the trees),

A phylogenetic analysis of the NS1 sequences of parvoviruses clearly demonstrates that the newly sequenced psittaciform chaphamaparvovirus 6 (PsChPV‐6) belongs to the genus *Chaphamaparvovirus*. As illustrated in Figure 1A, PsChPV‐6 is positioned within a distinct subclade of chaphamaparvoviruses, alongside other members of this genus identified from a range of host species. Notably, PsChPV‐6 exhibits a close phylogenetic relationship with chestnut teal chaphamaparvovirus 1, 2 and 3 (CTCPaV‐1, −2 and −3, GenBank accession nos MT247758‐60, MW046459, MW046575), as well as galliform chaphamaparvovirus 2 (GenBank accession no. MG846443).

In addition, a separate phylogenetic analysis based on the full genome sequence of beak and feather disease virus (BFDV) revealed that the BFDV genome sequenced in this study clusters within a distinct subclade. This subclade includes other BFDV strains previously isolated from Australian cockatoos (Figure 1B) and from nonpsittacine avian species, such as the white‐bellied sea eagle (*Haliaeetus leucogaster*) and peregrine falcon (*Falco peregrinus*). These findings emphasise the genetic diversity and host range of both PsChPV‐6 and BFDV, underlining the importance of continued surveillance and genetic characterisation of viral pathogens in wild and domestic bird populations.

## Discussion

The identification and genomic characterisation of a novel psittaciform chaphamaparvovirus 6 (PsChPV‐6) represents a significant contribution to our understanding of the genetic diversity and evolutionary dynamics of the *Chaphamaparvovirus* genus. The genome of PsChPV‐6, comprising 4232 nt and encoding major proteins, such as NS1 and VP1, exhibits key structural features that align it with other members of the *Parvoviridae* family.^19^, ^20^ This finding is consistent with the genomic composition of parvoviruses, characterised by high A + T content and the presence of multiple ORFs coding for critical viral proteins.^6^, ^19^, ^20^, ^21^ However, PsChPV‐6 also displays a number of unique genetic characteristics, particularly in the uncharacterised 5′ ORF1 region, which does not match known protein sequences. This novelty may indicate unrecognised functional elements or adaptations specific to this viral strain.

The comparative analysis of PsChPV‐6 NS1 and VP1 proteins demonstrates significant identity with other chaphamaparvoviruses, particularly psittacine parvovirus 1^7^ and various avian species‐specific parvoviruses. The conserved helicase and replication initiator motifs in the NS1 protein suggest that PsChPV‐6 retains essential replication machinery, like other parvoviruses.^20^ The identification of specific motifs such as Walker A, B and C further underscores the conserved nature of the replication mechanisms among parvoviruses while the variations seen in sequence identity point towards the virus's evolutionary divergence within its host species.^20^, ^22^, ^23^ The partial similarities with chestnut teal and galliform chaphamaparvoviruses emphasises the close evolutionary relationships within avian *Chaphamaparvovirus* species yet highlight potential host‐driven genetic divergence.

The evolutionary analysis presented in this study highlights the phylogenetic positioning of PsChPV‐6 within a distinct subclade of the *Chaphamaparvovirus* genus, alongside other strains isolated from a variety of bird species. The close relationship of PsChPV‐6 with chestnut teal chaphamaparvovirus 1, 2 and 3 suggests the existence of a shared evolutionary pathway among these viruses, likely driven by host‐specific factors.^20^, ^24^ This phylogenetic relationship also emphasises the importance of continued surveillance of parvoviruses across different avian species to better understand their genetic diversity, transmission dynamics and evolutionary drivers.

Beak and feather disease virus is a recognised and significant wildlife pathogen, primarily impacting psittacines, where it causes psittacine beak and feather disease (PBFD).^25^ Thought to have originated and coevolved among Australian parrots, BFDV's global spread has been exacerbated by the international trade of exotic psittacines, both legal and illegal.^26^, ^27^ The virus's persistence in the environment combined with its ability to switch hosts, has further accelerated its global distribution. This presents a substantial threat to global parrot conservation efforts, as BFDV's adaptability allows it to infect a broad range of bird species.^28^, ^29^, ^30^, ^31^, ^32^, ^33^ The BFDV identified in this study exhibits structural characteristics typical of the *Circoviridae* family^34^, ^35^ with phylogenetic clustering that links it to strains found in Australian cockatoos as well as nonpsittacine birds, including the white‐bellied sea eagle and peregrine falcon. This broad host range raises significant concerns about cross‐species transmission, indicating that BFDV poses a threat not only to psittacine birds but to a wider variety of avian species.^25^, ^30^, ^36^, ^37^, ^38^, ^39^

Overall, this study provides valuable insights into the genomic features and evolutionary relationships of PsChPV‐6 and BFDV. The high genetic diversity observed within PsChPV‐6, particularly in the uncharacterised ORF1 region, opens avenues for further functional studies to elucidate its role in viral replication and host interactions. Additionally, the close evolutionary relationships observed between PsChPV‐6 and other avian chapaparvoviruses underscore the need for ongoing monitoring of viral pathogens in avian populations. Future studies using systematic sampling across various avian species will be critical for improving our understanding of these viral pathogens and their role in avian health and disease.

## Conflicts of interest and sources of funding

The authors declare no conflicts of interest for the work presented here.

## Ethical approval

The faecal sampling was conducted during routine animal husbandry practice without touching animals. The Animal Ethics Committee at La Trobe University was informed that findings from the material were to be used in a publication, and a formal waiver of ethics approval was granted.

## Data Availability

The complete viral genome sequences from this study have been deposited in DDBJ/ENA/GenBank under the accession numbers OR729120 (link: https://www.ncbi.nlm.nih.gov/nuccore/OR729120) and OR729123 (link: https://www.ncbi.nlm.nih.gov/nuccore/OR729123). The version described in this paper is the first version, OR729120.1 and OR729123.1. The raw sequencing data from this study have been deposited in the NCBI Sequence Read Archive (SRA) under the accession number of SRR26413806 (link: https://www.ncbi.nlm.nih.gov/sra/SRR26413806), (BioProject accession number: PRJNA1028305, link: https://www.ncbi.nlm.nih.gov/bioproject/PRJNA1028305).
